# Health-related resources and social support as enablers of digital device use among older Finns

**DOI:** 10.1007/s10389-024-02241-y

**Published:** 2024-03-27

**Authors:** Johanna Eronen, Erja Portegijs, Taina Rantanen

**Affiliations:** 1 Faculty of Sport and Health Sciences and Gerontology Research Center, University of Jyväskylä, Jyväskylä, PO Box 35, 40014 Finland; 2 Center for Human Movement Sciences, University of Groningen, University Medical Center Groningen, Antonius Deusinglaan 1, 9713 AV Groningen, The Netherlands

**Keywords:** Technology, Aging, Health, Health literacy, Socioeconomic

## Abstract

**Aim:**

Older adults show considerable diversity in their use of digital devices, e.g., computers, tablets, and smart phones: some are non-users, some are learning to use them, and some use them fluently. The factors contributing to older adults’ digital device use are likely to differ between learners and fluent users. This paper examines whether different socioeconomic and health-related factors are associated with different levels of digital device use among older Finnish men and women.

**Subject and methods:**

Data from 750 community-dwelling men and women were collected with face-to-face interviews and postal questionnaires and analyzed using multinomial logistic regression analysis.

**Results:**

Younger age, presence of social support, and being partnered were associated with being a learner in digital device use, and higher education, a higher occupational status in working age, and higher health literacy were associated with being a fluent digital device user. Poor self-rated health was negatively associated with being a fluent user.

**Conclusions:**

Fluent digital device users have personal resources, such as higher education, good health, and high health literacy, that contribute to their digital skills. Social resources are important enablers for those learning to use digital devices.

## Introduction

The world is digitalizing and the significance of information technology in people’s everyday lives has increased rapidly over the past two decades. In 1999, 4% of Finnish persons aged 65 reported using a computer at least weekly (Official Statistics of Finland n.d.a). In 2002, the corresponding percentage was 15, with 5% reporting using the Internet at least weekly (Official Statistics of Finland n.d.a). In 2009, 33% of persons aged 65 reported having access to the Internet in their homes and 24% reporting using it at least once weekly (Official Statistics of Finland n.d.a). In 2021, 78% of persons aged 65 to 74 and 42% of persons aged 75 to 89 reported using the Internet daily or almost daily (Official Statistics of Finland n.d.b). Statistics from 2017 show that among 65- to 74-year-olds, 46% used the Internet for searching health-related information and 26% for making medical appointments, and 22% reported using social media at least once weekly (Official Statistics of Finland n.d.b). The corresponding percentages among persons aged 75–89 were 21, 9, and 8.

Despite the increase in digital device and Internet use, older adults continue to use information technology less and are more likely to experience problems in using digital devices and accessing the Internet than young or working-age persons. This digital divide has been identified not only between age groups but also within the older population: some older adults use information technology fluently in their everyday lives while others lack the ability, resources, and/-or interest required to use digital devices (Anderberg et al. 2020; Jørgensen et al. 2022). Previous studies have identified several factors that are associated with older persons using or not using digital devices. Digital device use has been associated in the general population with younger age, higher education, higher income, and better health (Quittschalle et al. 2020) and non-use with not feeling comfortable using a computer and not having the competence to use a computer (Arcury et al. 2018; Lee et al. 2018) and, especially among older people, with other factors such as reluctance, negative attitudes, lack of interest, lack of money, and/or not having anyone to encourage and help them (Anderberg et al. 2020; Luijkx et al. 2015). Some older adults report being cautious about the amount of time they spend using ICT, as they do not want it to distract them from other activities they consider more important (Nimrod 2020). The often-reported facilitators, such as having access to digital devices or the Internet at home, often co-exists with other facilitators such as younger age and higher education (Chang et al. 2015; Anderberg et al. 2020), and having someone to help with digital device use is often associated with a rich intergenerational network (Freeman et al. 2020). Negative attitude and lack of interest and comfort in computer use are more common among the oldest people and those with less education (Lee et al. 2018).

The terms digital divide and digital exclusion that are often used when discussing digital device use among older persons imply that that digital device use in this age group is a binary issue: one either uses or does not use these devices. Recently, however, it has been suggested that this division into users and non-users or those included and those excluded should be rethought, as many older persons categorized as non-users are in fact digital device users in one way or another (Anderberg et al. 2020; Gallistl et al. 2021). Moreover, becoming a digital device user is a process, prompted by interest and motivation, leading to learning, either alone or with support from other people, and culminating in the adoption, in various ways, of information technology in one’s everyday life (Wilson-Menzfeld et al. 2023). Thus, individuals’ digital device use can be considered a continuum, ranging from non-use to learning to use, possibly encountering some difficulty, and fluent use.

Studies have identified various factors associated with digital device use in old age. One approach to analyzing this association is to divide these factors into predisposing factors, need factors and enabling factors (Quittschalle et al. 2020). This categorization derives from Anderson’s behavioral model of health service use (Andersen 1995), which aimed at furthering understanding of why and how people use health care services by showing the contribution of different factors to service use. The Andersen model assumes that individual’s use of services is based on the socio-demographic characteristics and health beliefs (i.e., predisposing factors), experience of symptoms, illness, and pain (need factors) and personal and community resources (enabling factors) (Andersen 1995). The outcome, health service use, can be measured as use of different services or as access to care (Andersen 1995). The model has been widely used, discussed, and refined over the years (e.g., Linden et al. 1997; Lederle et al. 2021) and extended to other outcomes than health service use, such as health care costs (Heider et al. 2014) and barriers to care Imbus et al. 2018). Quittschalle et al. (2020) applied Anderson’s model in their study on older adults’ internet use. While we acknowledge that the determinants of health care service use and digital device or Internet use are unlikely to be the same, adopting this model facilitates examination of the factors associated with digital device use in a versatile way. We know that older adults use digital devices and the internet at different levels and volumes and for different purposes, such as finding information on health (Waterworth and Honey 2018), maintaining social activities (Aggarwal et al. 2020) or as compensation for a lack of social resources after loss of a partner, living far away from relatives, or when physical constrains hinder one from visiting friends (Nimrod 2020). Moreover, we know that the factors supporting older persons to become digital device users are various: some may attend a course (Juznic et al. 2006) while others receive support for purchasing and using a computer from their spouses, children, and grandchildren (Luijkx et al. 2015). Health-related factors, such as cognitive (Anderberg et al. 2020) and sensory functions may also play a role in this process.

In this study, we applied Anderson’s model as adapted by Quittschalle et al.’s (2020). The predisposing, need, and enabling variables were chosen based on previous studies which have adopted Andersen’s model and on factors which based on previous research are thought to influence digital device use. The predisposing variables were age, gender, education level, occupational attainment during working age, and perceived financial status. These sociodemographic factors are known to be associated with digital device use and feature in Andersen’s model. The need factors in this study were self-rated health, loneliness, depressive symptoms, and number of chronic conditions. These were chosen on grounds similar to those in the study by Quittschalle et al. (2020): they reflect health concerns. Finnish statistics show that searching for health-related information and checking one’s own information on patient portals are among the main reasons for Internet use by older adults (Statistics Finland n.d.). Loneliness may also encourage older adults to contact other people online (Gould et al. 2021). The enabling factors were personal and social resources. Social support and marital status were used in this study as indicators of social resources, as receiving support from family members and friends can be of critical importance in older adults’ uptake of technology. Finally, health literacy, which refers to the combination of competencies and resources that individuals need in order to access, understand, appraise, and use health-related information and services and make decisions about health (Sørensen et al. 2012), was used as an indicator of personal resources. Health literacy is a strong correlate of self-efficacy (Berens et al. 2022) and thus indicates the ability and confidence to cope with self-management.

This study aimed to answer the following research questions: What factors are associated with digital device use among older persons, and to what extent do they differ between those who do not use these devices at all, those who are learning to use them, and those who use them fluently?

## Methods

### Participants

This study used data collected in the “Active aging – resilience and external support as modifiers of the disablement outcome (AGNES)” cohort study conducted at the University of Jyväskylä, Finland (Rantanen et al., 2018). The data were collected in a structured home interview and with a postal questionnaire between October 2017 and December 2018, and with a postal questionnaire in April–June 2020. The 2020 postal questionnaire was an addition to the baseline, sent to the baseline participants during the state of emergency due to COVID-19, with the aim to investigate the consequences of the pandemic and social distancing recommendations among older adults. The AGNES cohort study baseline data (Rantanen et al., 2018) and the 2020 postal questionnaire (Rantanen et al., 2021) have been reported in detail elsewhere. In brief, the AGNES cohort study participants comprise a population-based sample of men and women aged 75, 80, and 85 at baseline, residing independently in the city of Jyväskylä in Central Finland. Those living in the study area, able to communicate, and willing to participate were invited to take part in the AGNES study. The initial baseline sample comprised 1021 persons. The 2020 postal questionnaire was sent to the 985 baseline participants who had not withdrawn their consent, of whom 809 responded.

The Ethical Committee of the Central Finland Health Care District approved the AGNES study on 23 August 2017 and the 2020 questionnaire on 13 May 2020. The Declaration of Helsinki has been followed throughout the AGNES study. All participants signed an informed consent form before participating in each phase of the study.

### Measures

Digital device use was measured with the postal questionnaire in 2020 (Eronen et al., 2021; Rantanen et al., 2021). The participants were asked to report whether they had difficulty in using digital devices, such as computers, tablets, or smart phones, with response options (1) no difficulty, (2) with minor difficulty, (3) with major difficulty, (4) unable without help, and (5) I don’t use any digital devices. For the analyses, these were grouped as fluent users (1), learners (2–4), and non-users (5).

Predisposing, need and enabling variables were assessed at the study baseline with home interviews and postal questionnaires. Predisposing variables included age group, gender, being partnered (married/cohabiting vs. divorced, widow, unmarried) education, occupation, and self-perceived financial status. Age and gender were drawn from the Finnish National Population Register. Information on marital status, as well as education, occupation, and self-perceived financial status were obtained via the study questionnaire. Education level was assessed by asking participants to report their highest education attainment, which was categorized as low (primary school or less), medium (middle school, folk high school, vocational school, or secondary school), or high (high school diploma or university degree). The participants were asked to report their longest-held occupation during working age. Occupations were categorized as manual, lower non-manual, and upper non-manual. Participants were asked to rate their perceived financial status as very good, good, satisfactory, or poor. For the analyses, the categories satisfactory and poor were combined.

The need factors included poor self-rated health, presence of depressive symptoms, loneliness, and number of chronic conditions. Self-rated health was assessed with a question on current general health with the response options very good, good, fair, poor, very poor. For the analyses, the categories very good/good and poor/very poor were combined. Depressive symptoms were assessed with the Center for Epidemiological Studies Depression Scale (CES-D, range 0–60) (Radloff 1977). The CES-D score was dichotomized into absence (0–16) and presence (> 16) of depressive symptoms (Sawyer Radloff and Teri 1986). Loneliness was asked with a question “How often do you feel lonely,” with response options very rarely or never, rarely, often, and almost always. For the analyses this was dichotomized as never/very rarely and at least sometimes (rarely, often, and almost always). Physician-diagnosed chronic conditions were self-reported during the home interview. Participants were asked to report whether they had any of the conditions listed under ten categories of chronic conditions and if so, to specify these. The ten categories were respiratory conditions, cardiac conditions, vascular conditions, cerebrovascular condition or brain injury, musculoskeletal condition, visual or auditory impairment, diabetes mellitus, malignant cancer, neurological conditions, and depression. In addition, participants were asked an open-ended question about any other physician-diagnosed chronic conditions they had, and their responses were later categorized by a nurse. For the present analyses, the number of chronic conditions each participant had was calculated by summing all the illnesses and conditions reported by the participants (range 0–12).

The enabling variables used in this study were social support and health literacy. Social support was assessed with three questions on the frequency of meeting children or other relatives, friends, and other acquaintances. The response options for these were (1) daily, (2) weekly, (3) monthly, (4) a few times a year, (5) only seldom, and (6) I do not have children/friends/acquaintances. Persons who met their children, friends, or acquaintances at least weekly were categorized as having social support. Health literacy was measured with the Finnish translation of the short form of the European Health Literacy Survey, HLS-EU-Q16 (Sørensen et al. 2013). Following the guidelines provided by the developers of the HLS-EU-Q, we calculated a health literacy score only for participants who had answered at least 80% of the items, i.e., given answers to at least 13 items in the questionnaire (Sørensen et al. 2013). The health literacy score was computed by recoding the response options very easy and easy as 1 and difficult and very difficult as 0. Summing the responses yielded a health literacy score ranging from 0 to 16, which in accordance with the guidelines issued by the developers of the instrument, was categorized as inadequate (0–8), problematic (9–12), and sufficient (13–16) health literacy, as recommended by the developers of the instrument (Sørensen et al. 2013).

The analyses were adjusted for baseline cognitive capacity, as assessed with the Mini-Mental State Examination (MMSE, range 0–30) (Folstein et al. 1975).

### Statistical analyses

Participant characteristics by category of digital device use are presented as percentages and means with standard deviations (SD). Associations of factors with digital device use were analyzed with multivariate logistic regression analyses. We report odds ratios (OR) with 95% confidence intervals (CI). We fitted four models: model 1 included age group, gender, education, occupation, and self-perceived financial status (predisposing variables); model 2 included poor self-rated health, loneliness, presence of depressive symptoms, and number of chronic conditions (need factors); and model 3 included social support, marital status, and health literacy (enabling variables). Model 4 included all statistically significant variables from models 1, 2, and 3 along with cognitive capacity. All analyses were performed with IBM SPSS version 28 and statistical significance was set at 0.05.

## Results

A total of 750 persons, of whom 58.4% were women, participated in the study. Participant characteristics by digital device use are presented in Table 1. More women were among the non-users than among fluent users (66.7% vs. 54.7%, *p* = 0.025). A higher percentage of the fluent users than non-users were aged 75 (59.7% vs. 29.1%) and a higher percentage of non-users than fluent users aged 85 (40% vs. 10.4%). Compared with non-users, fluent users more often had higher education (39.9% vs. 13.5%), had worked in upper non-manual positions (47.3% vs. 17.0%), and rated their financial status as very good (13.5% vs. 7.9%), all *p* < 0.001. Fluent users more often than non-users rated their health as good or very good (32.4% vs. 32.7%; *p* < 0.001) and were less likely to report the presence of depressive symptoms (9.7% vs.17.2%; *p* = 0.041) or feelings of loneliness (35.4% vs. 48.1%; *p* = 0.025). Learners and fluent users were more often partnered than non-users (64.7% and 65.6% vs. 42.0%; *p* < 0.001) and learners reported having more social support than fluent users or non-users (90.2% vs. 86.2% vs. 82.4%). These differences were not statistically significant (*p* = 0.054). The fluent user group showed the highest and the non-user group the lowest proportion of persons with sufficient health literacy (64.85% vs. 43.6%; *p* < 0.001). The fluent users showed the highest cognitive capacity (mean MMSE score 27.93, SD 1.86) and lowest mean number of chronic conditions (3.02, SD 1.92). The corresponding scores among the non-users were 26.52, SD 2.25 and 3.76, SD 2.06, *p* < 0.001.

**Table 1:** Participant characteristics by digital device use

	Non-users (*n* = 165)	Learners (*n* = 287)	Fluent users (*n* = 298)	*p*-value
	%	%	%	
Women	66.7	58.5	53.7	**0.025**
Age group				** < 0.001**
• 75	29.1	50.5	59.7	
• 80	30.9	36.6	29.9	
• 85	40.0	12.9	10.4	
Education				** < 0.001**
• Low	38.7	23.0	11.4	
• Intermediate	47.9	51.2	48.7	
• High	13.5	25.8	39.9	
Occupation				** < 0.001**
• Manual	33.9	23.7	9.7	
• Lower non-manual	48.5	40.1	40.3	
• Upper non-manual	17.0	34.8	47.3	
Self-perceived financial status				** < 0.001**
• Satisfactory/ poor	52.1	34.1	32.3	
• Good	40.0	54.7	54.2	
• Very good	7.9	11.1	13.5	
Self-rated health				** < 0.001**
• Good/very good	32.7	51.2	62.4	
• Satisfactory/ Poor	67.3	48.8	37.6	
Presence of depressive symptoms	17.2	17.5	9.7	**0.014**
Feelings of loneliness	48.1	41.8	35.4	**0.025**
Partnered	42.0	65.6	64.7	** < 0.001**
Has social support	82.4	90.2	86.2	0.054
Health literacy level				** < 0.001**
• Sufficient	43.6	53.3	64.8	
• Problematic	37.0	34.5	28.9	
• Inadequate	19.4	12.2	6.4	
	mean (SD)	mean (SD)	mean (SD)	
MMSE	26.52 (2.52)	27.61 (1.96)	27.93 (1.86)	** < 0.001**
Number of chronic conditions	3.76 (2.06)	3.43 (2.09)	3.02 (1.92)	** < 0.001**

The results of the multinomial logistic regression analyses are presented in Table 2. Model 1 included the predisposing variables, i.e., age group, gender, education, occupation, and perceived financial status. Comparison of learners with non-users revealed that younger age was associated with higher odds for being a learner than a non-user. Younger age, male gender, higher education, and higher occupational status during working age were associated with a higher likelihood of being a fluent user than a non-user. The only need-related variable (model 2) associated with digital device use was self-rated health: the odds for poor self-rated health were lower among the learners (OR 0.49, 95% CI 0.32–0.76) and fluent users (OR 0.37, 95% CI 0.24–0.58) than non-users. Of the enabling variables, having social support, being partnered, and having sufficient health literacy were positively associated with being a learner vs. being a non-user, and being partnered and having higher health literacy were positively associated with being a fluent user vs. a non-user.

**Table 2:** Predisposing, needs, and enabling factors and their association with digital device use

	Model 1				Model 2				Model 3				Model 4			
	Learners vs. non-users		Fluent users vs. non-users		Learners vs. non-users		Fluent users vs. non-users		Learners vs. non-users		Fluent users vs. non-users		Learners vs. non-users		Fluent users vs. non-users	
	OR	95% CI	OR	95% CI	OR	95% CI	OR	95% CI	OR	95% CI	OR	95% CI	OR	95% CI	OR	95% CI
*Predisposing*																
Age group																
75	**4.86**	**(2.85–8.29)**	**6.76**	**(3.82–11.96)**									**4.01**	**(2.28–7.05)**	**5.12**	**(2.80–9.34)**
80	**3.46**	**(2.02–5.93)**	**3.49**	**(1.94–6.26)**									**3.04**	**(1.73–5.34)**	**3.03**	**(1.63–5.61)**
85	1		1										1		1	
Gender																
Male	1.27	(0.81–1.98)	**1.73**	**(1.09–2.75)**									1.15	(0.70–1.90)	1.59	(0.95–2.66)
Female	1		1										1		1	
Education																
High	1.62	(0.72–3.61)	**3.74**	**(1.63–8.56)**									1.31	(0.57–3.04)	**3.02**	**(1.27–7.16)**
Intermediate	1.37	(0.83–2.27)	**2.09**	**(1.18–3.70)**									1.41	(0.84–2.37)	**2.22**	**(1.23–4.01)**
Low	1		1										1		1	
Occupation																
Upper non-manual	1.79	(0.85–3.78)	**3.92**	**(1.77–8.70)**									1.80	(0.83–3.92)	**3.69**	**(1.62–8.43)**
Lower non-manual	1.08	(0.64–1.83)	**2.49**	**(1.35–4.59)**									1.09	(0.63–1.88)	**2.39**	**(1.28–4.50)**
Manual	1		1										1		1	
Self-perceived financial status																
Very good	1.50	(0.70–3.24)	1.39	(0.64–3.02)												
Good	1.53	(0.98–2.38)	1.29	(0.81–2.06)												
Satisfactory/ poor	1		1													
*Needs*																
Self-rated health																
Satisfactory/ poor					**0.49**	**(0.32–0.76)**	**0.37**	**(0.24–0.58)**					0.72	(0.46–1.13)	**0.53**	**(0.33–0.84)**
Very good/ good					1		1						1		1	
Loneliness																
Yes					0.81	(0.54–1.22)	0.70	(0.46–1.05)								
No					1		1									
Depressive symptoms																
Yes					1.38	(0.80–2.37)	0.84	(0.46–1.52)								
No					1		1									
Chronic conditions	0.98	(0.89–1.08)	0		0.98	(0.89–1.08)	0.92	(0.83–1.02)								
*Enabling*																
Social support																
Yes									**1.96**	**(1.10–3.48)**	1.26	(0.73–2.17)	**2.10**	**(1.12–3.95)**	1.55	(0.83–2.92)
No									1		1		1		1	
Marital status																
Partnered									**2.59**	**(1.74–3.85)**	**2.40**	**(1.61–3.57)**	**1.89**	**(1.18–3.02)**	1.56	(0.95–2.53)
Unmarried									1		1		1		1	
Health literacy																
Sufficient									**1.86**	**(1.05–3.29)**	**4.35**	**(2.30–8.23)**	1.30	(0.69–2.44)	**2.60**	**(1.27–5.32)**
Problematic									1.56	(0.86–2.81)	**2.45**	**(1.26–4.77)**	1.45	(0.76–2.77)	**2.24**	**(1.06–4.71)**
Inadequate									1		1		1		1	

**Fig. 1:**
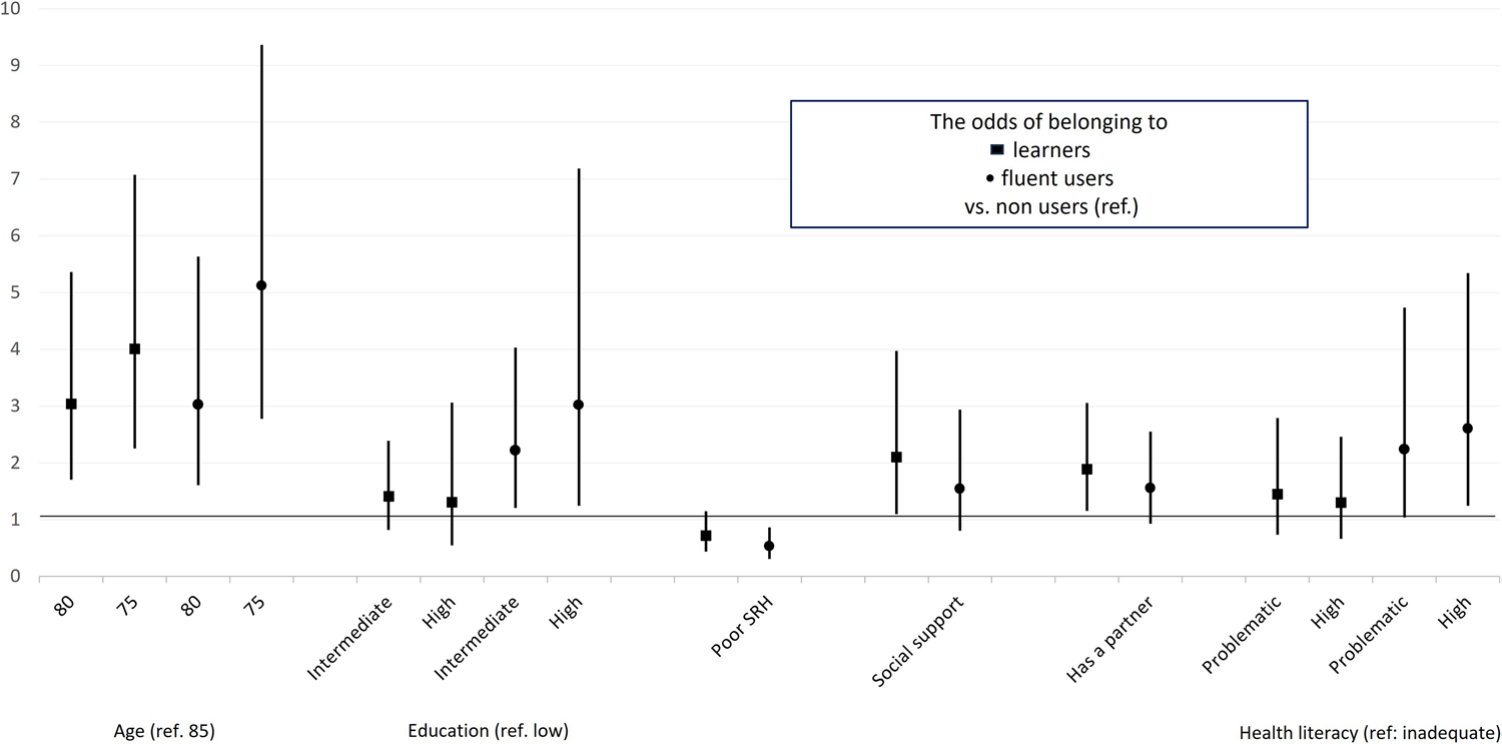
The association of age group, education level, poor self-rated health (SRH), presence of social support, having a partner, and health literacy with belonging to learners and fluent users vs. non-users. Odds ratios with 95% confidence intervals. If the confidence interval is above or below 1, the result is statistically significant

When all the statistically significant variables from models 1–3 were entered into model 4 (Fig. 1), younger age was found to be associated with higher odds for being a learner than a non-user (OR 4.01, 95% CI 2.28–7.05 for 75-year-olds and OR 3.04, 95% CI 1.73–5.34 for 80-year-olds). In addition, having social support (OR 2.10, 95% CI 1.12–43.95) and being partnered (OR 1.89, 95% CI 1.18–3.02) were associated with a higher likelihood of being a learner than a non-user. Comparison of fluent users with non-users showed that in addition to younger age, higher education (OR 3.02, 95% CI 1.27–7.16), higher occupational status during working age (3.69, 95% CI 1.62, 8.43), and higher health literacy (OR 2.60, 95% CI 1.27–7.16) were more strongly associated with membership of the fluent user than non-user group. Finally, poor self-rated health was associated with lower odds for fluent digital device use (OR 0.53, 95% CI 0.33–0.84).

## Discussion

This study examined older adults’ digital device use by comparing persons who were fluent digital device users and persons who reported using such devices with some degree of difficulty, here termed learners, with those who reported being non-users. A novel finding was that the factors associated with being a learner differed from those associated with being a fluent user or a non-user. The results showed that digital device use was more common among the youngest study participants, i.e., the 75-year-olds. In addition, being a learner was associated with having social support and being partnered. In turn, being a fluent user was associated with higher education, higher occupational status during working age, better self-rated health, and higher health literacy.

Thus, different factors were found to contribute to digital device use, and the so-called digital divide, in the present sample of older persons. This result confirms previous findings that, among older adults, the use of digital devices and the Internet is a more likely option for those who have better financial, social, and health resources (Din et al. 2019). To put it bluntly, it is arguable that access to digital devices and spaces is the privilege of the more affluent. From the health standpoint, it seems that older adults who have fewer health issues also have the best access to digital health information. While the causality of this finding may be questioned, it is nevertheless cause for concern that older persons with poorer health more often lack digital opportunities to find information about their illnesses or health care providers, or to manage their own health records in patient portals.

Older persons are thus heterogeneous in their use of digital devices. In today’s rapidly digitalizing world, fewer and fewer older adults are any longer non-users of digital technology, as it has already been incorporated into many daily activities. In fact, it may soon become impossible to conduct normal everyday life without using any digital devices at all. Moreover, our results, which focused on computer and smart phone use, showed that social support increased the odds for their use by non-fluent users. Previous studies have shown that living with someone or having a friend or relative who can use a computer, etc., lowers the threshold for trying to learn how to use digital devices and eHealth technology (Airola 2021). Typically, older persons receive support for digital device use from their spouses, children, and grandchildren (Juznic et al. 2006; Luijkx et al. 2015). Studies on gender differences in digital device use have generally found that older men are more often Internet users (Anderberg et al. 2020) and have a higher interest and are more comfortable in using computers than women (Lee et al. 2018). In our study, men were also more likely to be fluent uses than women, although gender did not play a role in the final model.

Having a higher education and having worked in non-manual positions were strongly associated with fluency in the use of digital devices. This confirms previous findings (Anderberg et al. 2020; Lee et al. 2018). For some of the participants who reported being fluent users, their previous occupations may have required them to use digital devices. Apart from work history, higher socioeconomic status likely indicates better resources for purchasing digital devices and predict more active participation in adult education programs aimed at improving digital skills (Heng Wen Ngiam et al. 2022; Juznic et al. 2006). There is also evidence that higher education predicts interest in and feeling comfortable with the use of computers (Lee et al. 2018).

In our study, better self-rated health was associated with being a fluent digital device user. This finding points to the importance of our inclusion of self-rated health as a need variable in the present analyses. Our reason for this was that, given the importance of the Internet as a source of health-related information, having concerns about one’s health would motivate digital device use (Nguyen et al. 2017). However, our result could also be interpreted as meaning that better self-rated health and a lower number of chronic conditions are facilitators of digital device use and, hence, according to the Andersen model, an enabling variable. There is evidence to support this: better health is associated with a higher likelihood of Internet use (Tavares 2020), and health-related difficulties and frailty are the most frequently reported barriers to computer and eHealth use (Airola 2021; Jørgensen et al. 2022). Our results also showed that higher health literacy was associated with being a fluent digital device user. Health literacy can indicate better self-efficacy (Berens et al. 2022), which may in turn influence the motivation for learning and using digital devices (Jokisch et al. 2022). Access to digital spaces and the Internet can also have a positive impact on health literacy (Mackert et al. 2016). However, in our study, as health literacy was assessed at baseline and digital device use at follow-up, and we know nothing about the participants digital device use at or prior to the baseline, we cannot draw conclusions about the temporal order of these two variables. It is nevertheless likely that high health literacy and the ability to use computers and smart phones have similar correlates, such as higher socioeconomic status and self-efficacy (Jokisch et al. 2022).

The ability to use a computer or a mobile device and the opportunity to access the Internet enable greater autonomy in many activities of everyday life, such as banking, social relations, and information-seeking for different purposes. Although the proportion of older adults who are digital device users is constantly increasing, it needs to be remembered that computer users among persons aged 80 and over continue to be in the minority (Anderberg et al. 2020). Findings that older persons are willing and able to learn to use information technology are, however, encouraging (Arthanat 2019). Our results confirm this and indicate that efforts to increase the number of digital device users among older adults should target, in particular, those who live alone, have health problems, and whose socioeconomic status is low. While the Internet can be a powerful tool in making, for example, health-related information accessible to wider audiences, the risk remains that this information and the applications that distribute it, such as patient portals, are in fact only available to those with better economic, health, and social resources. Service providers must guarantee that information and services are accessible to all, regardless of their digital proficiency. The importance of imparting digital skills to older adults remains, given the continuous advancement and evolution of technology, along with the introduction of new services and devices. Looking ahead, it is crucial to involve older adults in the co-creation process when planning and developing digital services, to ensure they are user-friendly. Moreover, future research exploring digital device usage should consider including the oldest age groups in their interventions.

### Strengths and limitations

This study used data from a relatively large population-based sample. However, it has its limitations. First, the participants were relatively healthy older adults. Second, we did not have information on what digital devices the participants used or on their reasons for using these devices. Third, we do not know how many of those who reported using digital devices used them for browsing the Internet.

## Conclusion

This study demonstrated that age, social support, being partnered, education, occupational status in working age, health literacy, and self-rated health were associated with digital device use among older persons. More specifically, younger age, presence of social support, and being partnered were associated with being a learner in digital device use, and higher education, a higher occupational status in working age, better self-rated health, and higher health literacy were associated with being a fluent digital device user.

## Data Availability

After completion of the AGNES study, data will be stored at the Finnish Social Science Data Archive without potential identifiers (open access). Until then, pseudonymized datasets are available to external collaborators upon agreement on the terms of data use and publication of results. To request data, please contact Professor Taina Rantanen (taina.rantanen@jyu.fi).
